# Topical C-Phycocyanin-Loaded Transfersomes Attenuate Early Proinflammatory Epidermal Remodelling in a DMBA/TPA-Induced Mouse Model of Skin Dysplasia

**DOI:** 10.3390/pharmaceutics18050600

**Published:** 2026-05-14

**Authors:** Daiva Galinytė, Nomeda Juodžiukynienė, Ingrida Balnytė, Vilma Zigmantaitė, Jūratė Karosienė, Jurga Bernatoniene, Nijolė Savickienė

**Affiliations:** 1 Department of Pharmacognosy, Faculty of Pharmacy, Academy of Medicine, Lithuanian University of Health Sciences, Sukilėlių av. 13, 50162 Kaunas, Lithuania; nijole.savickiene@lsmu.lt; 2 Department of Veterinary Pathobiology, Faculty of Veterinary, Academy of Veterinary, Lithuanian University of Health Sciences, Tilžės St. 18, 47181 Kaunas, Lithuania; nomeda.juodziukyniene@lsmuni.lt; 3 Department of Histology and Embryology, Faculty of Medicine, Academy of Medicine, Lithuanian University of Health Sciences, A. Mickevičiaus St. 9, 44307 Kaunas, Lithuania; ingrida.balnyte@lsmu.lt; 4 Biological Research Centre, Lithuanian University of Health Sciences, Tilžės St. 18, 47140 Kaunas, Lithuania; vilma.zigmantaite@lsmu.lt; 5 Laboratory of Membrane Biophysics, Institute of Cardiology, Lithuanian University of Health Sciences, Sukilėlių av. 15, 47181 Kaunas, Lithuania; 6 Laboratory of Algology and Microbial Ecology, State Scientific Research Institute, Nature Research Centre, Akademijos St. 2, 08412 Vilnius, Lithuania; jurate.karosiene@gamtc.lt; 7 Department of Drug Technology and Social Pharmacy, Faculty of Pharmacy, Academy of Medicine, Lithuanian University of Health Sciences, Sukilėlių av. 13, 50162 Kaunas, Lithuania; jurga.bernatoniene@lsmu.lt

**Keywords:** c-phycocyanin, transfersomes, topical delivery, skin cancerogenesis, DMBA/TPA model, cutaneous squamous cell carcinoma, chemoprevention, Ki-67 proliferation index

## Abstract

**Background/Objectives**: Cutaneous squamous cell carcinoma (cSCC) develops through inflammation-driven preneoplastic alterations characterized by epidermal hyperplasia, dysplasia, and increased proliferative activity. C-phycocyanin (C-PC) possesses antioxidant and anti-inflammatory properties; however, its topical potential to attenuate a tumour-promoting cutaneous microenvironment is limited by poor skin penetration. This study evaluated the effects of C-PC-loaded transfersomes in a 7,12-dimethylbenz[*a*]anthracene (DMBA)/12-O-tetradecanoylphorbol-13-acetate (TPA)-induced mouse model of skin carcinogenesis. **Methods**: Male BALB/c mice were assigned to six groups (n = 10 per group). Carcinogenesis was initiated with a single topical application of DMBA, followed by twice-weekly TPA application for 16 weeks. C-PC-loaded transfersomes (1 mg/mL or 10 mg/mL) were applied topically. Histopathological assessment included epidermal thickness, rete ridge depth, mitotic activity, mast cell density, and semi-quantitative scoring of hyperplasia, dysplasia, and inflammation. Ki-67 immunohistochemistry was used to evaluate basal and suprabasal proliferation. **Results**: Carcinogen exposure induced marked epidermal thickening, severe dysplasia, increased mitotic activity, elevated Ki-67 expression, and pronounced dermal inflammation. Treatment with C-PC-loaded transfersomes significantly reduced epidermal thickness, rete ridge depth, mast cell density, mitotic counts, and suprabasal Ki-67 index. The 1 mg/mL concentration demonstrated the most consistent attenuation of dysplasia severity and inflammatory changes. No adverse histopathological alterations were observed in internal organs. **Conclusions**: These findings indicate that transfersome-mediated topical delivery of C-PC attenuates early inflammation-driven epidermal remodelling and tumour-promoting alterations in experimental skin carcinogenesis, supporting its potential as a topical preventive strategy.

## 1. Introduction

cSCC is one of the most common non-melanoma skin cancers and represents a growing public health concern due to its steadily increasing incidence worldwide [1]. The disease originates from an uncontrolled proliferation of atypical epidermal keratinocytes and is generally considered the result of a prolonged intraepidermal dysplastic process [2]. cSCC typically develops through a multistep progression from actinic keratosis (AK), a precancerous lesion characterized by dysplastic keratinocytes, to invasive carcinoma [2]. Ultraviolet radiation (UVR)-induced DNA damage, chronic inflammation, and local immunosuppression are central drivers of this process, leading to genetic and epigenetic alterations that disrupt epidermal homeostasis and promote pathological keratinocyte proliferation. Major risk factors include advanced age, cumulative UVR exposure, fair skin, male sex, immunosuppression, smoking, and genetic susceptibility, while human papillomavirus infection may further contribute to the development of the specific anatomical sites [1,2]. Despite advances in surgical and non-surgical management, cSCC incidence and associated morbidity continue to rise, underscoring the importance of preventive strategies targeting early, preinvasive stages of disease development.

Given that cSCC arises from a prolonged precancerous phase characterized by oxidative stress, chronic inflammation, and progressive disruption of the epidermal microenvironment, therapeutic intervention at early stages is particularly attractive. However, current preventive approaches, including UVR protection, provide only partial efficacy and are often limited by compliance, tolerability, or inconsistent clinical benefit [1]. Therefore, increasing attention has been directed toward identifying safe, biologically active compounds that can modulate key pathogenic mechanisms before irreversible malignant transformation occurs.

Natural compounds with antioxidant and anti-inflammatory properties are particularly promising in this context. Various phytochemicals, including plant polyphenols such as resveratrol, curcumin, catechins, quercetin, and honokiol have been widely investigated as chemopreventive agents against skin carcinogenesis due to their antioxidant, anti-inflammatory, DNA-protective, and antiproliferative properties [3]. However, despite promising preclinical findings, their clinical applications remain limited by poor bioavailability, rapid metabolism, and insufficient skin penetration, necessitating the development of advanced delivery strategies, including nanoengineered delivery systems to enhance their pharmacokinetic features. Moreover, there is still insufficient clinical evidence to confirm their efficacy and long-term safety [3,4]. Among existing chemopreventive approaches, retinoids represent one of the most extensively studied and clinically applied classes of agents for non-melanoma cancers, including cSCC. These compounds regulate key processes in carcinogenesis, including cell proliferation, differentiation, and inflammation. However, their clinical use remains limited due to adverse effects, including hepatotoxicity and metabolic disturbances, as well as emerging evidence of resistance development in cancer cells [5].

In this context, C-PC, a bioactive phycobiliprotein derived from cyanobacteria, has attracted increasing attention for its multifunctional biological activity. C-PC has demonstrated potent antioxidant, anti-inflammatory, and anticancer effects in multiple experimental models [6,7]. Accumulating evidence indicates that C-PC exerts protective effects against various skin disorders through its strong antioxidant capacity, enabling efficient scavenging of reactive oxygen species (ROS) and attenuation of oxidative stress-driven inflammation and carcinogenic processes [8,9]. In addition, C-PC has been shown to mitigate UVB-induced skin damage and delay photoaging by suppressing matrix metalloproteinase activity while preserving the expression of key epidermal barrier proteins, including involucrin, filaggrin, and loricrin [10]. Furthermore, C-PC inhibits melanoma cell proliferation and migration by modulating key regulators of tumour progression, including MAPK-associated signalling pathways, cell cycle regulators (CDK4/6), the angiogenic factor VEGF, the cell adhesion molecule N-cadherin, and matrix metalloproteinase MMP-9. Importantly, these effects are predominantly cytostatic and do not compromise the viability of non-tumorigenic cells [11]. Collectively, these findings suggest that C-PC can interfere with early tumour-promoting pathways by limiting cell proliferation and migration through modulation of signalling molecules involved in cell cycle regulation, angiogenesis, and extracellular matrix remodelling, while maintaining a favourable safety profile [11,12,13]. However, despite these promising properties, the topical preventive potential of C-PC against cSCC development remains largely unexplored, particularly in vivo. Moreover, due to its high molecular weight, topical formulations containing C-PC face significant challenges in achieving adequate skin penetration. Transfersomal systems are particularly suited to overcoming this limitation due to their high membrane elasticity, which enables passage through intercellular skin pathways that are smaller than their vesicle diameter. Importantly, the biological performance of such systems depends not only on drug loading but also on the vesicle deformability, physicochemical stability, and skin deposition properties. In our previous study, we developed C-PC-loaded glycerol-transfersomes designed to enhance epidermal delivery of this bioactive compound and demonstrated their cytocompatibility and cytoprotective effects against oxidative stress in HaCaT keratinocytes, thereby providing a rationale for subsequent in vivo evaluation [14]. We hypothesized that improved topical delivery of C-PC protects against early-stage cSCC by reducing oxidative stress, inflammation, and pathological epidermal proliferation. To test this hypothesis, the present study employed a DMBA/TPA-induced mouse model of skin carcinogenesis, which distinguishes between tumour initiation and promotion phases and is widely used to evaluate chemopreventive strategies [15]. Importantly, DMBA/TPA-induced tumours show similarities to human cSCC, including alterations in oncogenic signalling pathways and the involvement of inflammation-driven tumour progression [16,17].

## 2. Materials and Methods

### 2.1. Materials

C-PC was isolated from non-toxic cyanobacterial biomass harvested from the Kaunas Reservoir (Kaunas, Lithuania) and the Simnas fishpond (Simnas, Lithuania), characterized by the dominance of *Aphanizomenon flos-aquae* (>95% of total phytoplankton biomass), as previously described by Galinytė et al. [14]. The purity of the obtained C-PC was 3.227 ± 0.045 (OD620/OD280) [14].

C-PC-loaded glycero-transfersomes (T-PC) were prepared and characterized as previously described by Galinytė et al. [14]. The selected formulation was characterized by a nanoscale vesicle size (~100 nm), a narrow polydispersity index (<0.2), a high negative zeta potential (≤−30 mV), high C-PC encapsulation efficiency (~50%), and predominantly spherical unilamellar vesicles confirmed by cryo-transmission electron microscopy [14].

DMBA, TPA and Mayer’s hematoxylin were purchased from Sigma-Aldrich (St. Louis, MO, USA). Anti-Ki67 antibody (ab16667, clone SP6) was purchased from Abcam (Cambridge, UK). DCS LabLine Antibody Dilution Buffer (AL120R100), DCS DetectionLine SUPERVision-2 Polymer-Reagent (HRP, LD521R125), citrate buffer (pH 6, CL009C500), and DCS LabLine IHC Wash Buffer (WL583C2500) were obtained from DCS Innovative Diagnostik-Systeme (Hamburg, Germany).

### 2.2. In Vivo Experimental Design

The DMBA/TPA-induced mouse skin tumorigenesis protocol was performed as previously described by Li et al. [17]. The potential of topical C-PC-loaded transfersomes to modulate a tumour- promoting cutaneous microenvironment was evaluated.

In vivo experiments were performed in 2025 at the Lithuanian University of Health Sciences Biological Research Centre (Kaunas, Lithuania). All animal experiments were approved by the State Food and Veterinary Service of the Republic of Lithuania (approval No. G2-236) and conducted in accordance with national and European Union legislation.

Male BALB/c albino mice (*Mus musculus*), aged 8–12 weeks, were obtained from the Lithuanian University of Health Sciences Vivarium (Kaunas, Lithuania). Animals were acclimatized for 3 days before the study and housed under controlled environmental conditions (ambient temperature of 21 ± 2.5 °C and relative humidity of 50–55%; 12 h light/12 h dark cycle), with *ad libitum* access to water and food (standard pelleted rodent diet).

Animals were randomly assigned to six groups (n = 10 per group). The dorsal skin was shaved once per week using an electric clipper. The experimental protocol consisted of an initiation–promotion scheme using DMBA and TPA. Skin carcinogenesis was initiated by a single topical application of DMBA (50 µg in acetone). One week later, tumour promotion was carried out by topical application of TPA (5 µg in acetone or PEG/acetone solution) twice per week for 16 weeks. Topical treatment with C-PC-loaded transfersomes (T-PC) or empty transfersomes was applied twice per week, 2 h after each TPA application. All formulations were applied at a fixed dose of 200 µL per animal to a defined dorsal skin area (2 × 2 cm). All carcinogen-exposed groups received identical DMBA/TPA treatment and differed only in the type of topical formulation applied (C-PC-loaded transfersomes at different concentrations, empty transfersomes, or no additional treatment). In addition, separate non-carcinogen groups were included; these were not exposed to DMBA/TPA and received topical application of C-PC-loaded transfersomes at different concentrations. The detailed treatment protocol is presented in Table 1. The selected concentrations of C-PC (1 and 10 mg/mL) were based on previously published studies reporting wide ranges of effective concentrations, depending on the experimental model and route of administration. For example, topical C-PC formulations at ~2.5 mg/mL have been shown to exert protective effects in UVB-induced skin damage models, while concentrations of 2–6 mg/mL demonstrated biological activity in wound healing models [18,19]. Considering this variability and the limited skin penetration of high-molecular-weight compounds, a relatively broad concentration range was selected to evaluate potential dose-dependent effects.

Animals were weighed twice weekly, and routine clinical observations were performed throughout the study. After 16 weeks, animals were euthanized by cervical dislocation, in accordance with institutional and European Union guidelines. Skin and internal organ samples were collected and fixed in 10% neutral buffered formalin for further analysis.

### 2.3. Histotechnique

Skin and internal organ specimens (liver, spleen, lungs, heart, kidneys, brain, testes, mesenteric lymph node) from each animal were collected for histopathological examination. The samples were fixed in 10% neutral-buffered formalin at room temperature. Paraffin blocks were prepared using a Shandon Pathcentre embedding system (Thermo Fisher Scientific, Waltham, MA, USA) and a TES 99 tissue processor (Medite Medizintechnik GmbH, Bergdorf, Germany). Serial 4-μm sections from each sample were cut using a Sakura Accu-Cut SRM microtome (Sakura Finetek, Torrance, CA, USA) and stained with routine hematoxylin and eosin (H&E).

### 2.4. Histopathological Examination

Histological slides were evaluated, and image analysis was performed using a light microscope (Olympus BX41, Olympus Corporation, Tokyo, Japan) equipped with a DP72 digital camera and CellSens Dimension software version 1.16 (Olympus Corporation, Tokyo, Japan). A predefined set of epidermal and dermal histomorphological parameters was assessed. Epidermal criteria included epidermal thickness, rete ridge depth, hyperplasia, dysplastic alterations, mitotic activity, and spongiosis, whereas dermal criteria comprised inflammatory infiltrate, circulatory changes, and mast cell density. Semiquantitative alterations were graded on a 4-point scale (0 = absent, 1 = mild, 2 = moderate, 3 = severe), while quantitative parameters were expressed as measurements (µm) or absolute counts per standardized area (2.37 mm^2^).

Histopathological evaluation was performed to assess the potential systemic toxicity of the tested formulations. Organ-specific histomorphological examination was conducted in the heart, liver, kidneys, spleen, lungs, testes, and mesenteric lymph nodes to identify degenerative, necrotic, inflammatory, adaptive, and neoplastic alterations.

### 2.5. Immunohistochemistry: Ki-67

Immunohistochemistry for Ki-67 was performed on all skin samples. Formalin-fixed, paraffin-embedded tissue sections were mounted on poly-L-lysine-coated slides (Thermo Fisher Scientific, Branchburg, NJ, USA). Heat-induced antigen retrieval was carried out in citrate buffer (pH 6) (CL009C500; DCS Innovative Diagnostik-Systeme, Hamburg, Germany) for 30 min at 110 °C using a pressure cooker (Thermo Fisher Scientific, Branchburg, NJ, USA). The Shandon CoverPlate System (Thermo Fisher Scientific, Branchburg, NJ, USA) was used for staining. Endogenous peroxidase activity was quenched with 3% hydrogen peroxide solution (Valentis, Kaunas, Lithuania) and then further blocked with Hydrogen Peroxide Blocking Reagent (AB64218; Abcam, Cambridge, UK). Primary Rb mAb to Ki67 antibody (ab16667, clone SP6; Abcam, Cambridge, UK) was applied at a 1:200 dilution and incubated overnight at 4 °C. DCS LabLine Antibody Dilution Buffer (AL120R100; DCS Innovative Diagnostik-Systeme, Hamburg, Germany) was used for antibody dilution. Slides were subsequently incubated with DCS DetectionLine SUPERVision-2 Polymer-Enhancer (LD511R125; DCS Innovative Diagnostik-Systeme, Hamburg, Germany) for 30 min at room temperature, followed by the DCS DetectionLine SUPERVision-2 Polymer-Reagent (HRP) (LD521R125; DCS Innovative Diagnostik-Systeme, Hamburg, Germany) for 30 min at room temperature. Immunoreactivity was visualized using DCS ChromoLine DAB concentrate (DC136C007; DCS Innovative Diagnostik-Systeme, Hamburg, Germany) diluted in DCS ChromoLine DAB substrate buffer (PC137R125; DCS Innovative Diagnostik-Systeme, Hamburg, Germany) at a ratio of 1 drop per 1 mL, applied for 4 min at room temperature. DCS LabLine IHC Wash-Buffer (WL583C2500; DCS Innovative Diagnostik-Systeme, Hamburg, Germany) was used after each step. Slides were counterstained with Mayer’s hematoxylin solution (Sigma -Aldrich, Taufkirchen, Germany), dehydrated and mounted.

Ki-67 expression was evaluated separately in the basal and suprabasal (parabasal) layers of the epidermis. For each animal, 10 non-overlapping digital images at 200× magnification were analysed in the most representative hyperplastic or dysplastic areas, avoiding ulcerated and heavily crusted regions. In each image, all basal and parabasal keratinocytes were counted, and the number of Ki-67-positive nuclei was recorded. The Ki-67 index was calculated as the percentage of positive cells relative to the total number of basal or parabasal cells, respectively.

### 2.6. Statistical Analysis

Statistical analysis was performed using IBM SPSS Statistics version 29.0 (IBM, Armonk, NY, USA). Data distribution normality was assessed using the Shapiro–Wilk test. Homogeneity of variances was evaluated using Levene’s test.

For normally distributed data with homogeneous variances, one-way ANOVA followed by Tukey’s post hoc test was applied. When the homogeneity of variance assumptions were violated, Welch’s ANOVA followed by the Games–Howell post hoc test was used. Non-normally distributed variables and ordinal data (e.g., hyperplasia, dysplasia, inflammation scores) were analysed using the Kruskal–Wallis test with Bonferroni-adjusted pairwise comparisons.

Correlation analyses between proliferative indices and histopathological parameters were performed using Spearman’s rank correlation coefficient (ρ). A *p*-value < 0.05 was considered statistically significant.

## 3. Results

### 3.1. General Observations on the Effects of C-PC-Loaded Transfersomes on Skin Tumour Development and Animal Well-Being

Topical application of DMBA followed by repeated TPA treatment induced visible skin alterations in mice, including erythema, hyperkeratosis, and papilloma-like lesions, indicating early-stage activation of the tumorigenesis process.

Animals were monitored daily for health and welfare status and twice weekly for body weight and general clinical condition, using predefined animal welfare scoring criteria. During the initial tumour promotion phase (weeks 1–5), TPA was administered in acetone as a vehicle, resulting after approximately 4–5 weeks in pronounced local skin irritation in all TPA-treated groups. Increased scratching behaviour led to superficial skin lesions predominantly located in areas accessible to scratching or grooming rather than at the direct application site.

To reduce animal discomfort and comply with animal welfare requirements, the TPA acetone vehicle was modified to a TPA PEG/acetone solution from the 6th to the 12th week of treatment. This modification markedly alleviated skin irritation scores and reduced self-inflicted skin lesions. During the final experimental phase (weeks 13–16), TPA in acetone was reintroduced to further enhance carcinogenesis promotion; however, despite the intensified promotion protocol and adherence to animal welfare requirements, fully developed squamous cell carcinoma could not be induced.

Throughout the experimental period, animals remained active, with normal posture and locomotion, and no signs of impaired respiration, systemic distress, or treatment-related mortalities were observed.

**Figure 1:**
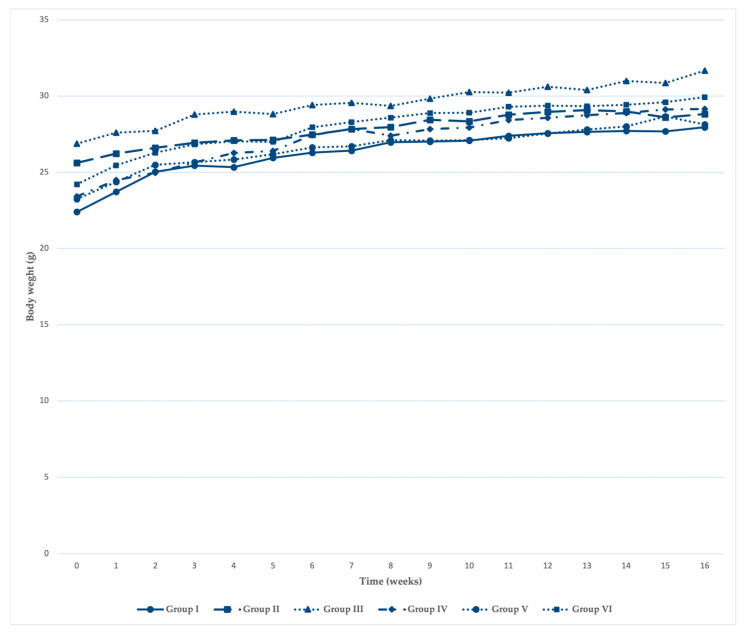
Body weight monitoring during the experimental period.

Body weight analysis demonstrated a significant effect of time on body weight (*p* < 0.001), reflecting normal growth (Figure 1), as well as significant time × group interaction (*p* = 0.004) and group effects (*p* = 0.001); however, body weight values remained within physiological ranges, with no signs of systemic toxicity.

Visually assessed composite skin irritation scores (including erythema, hyperpigmentation, dryness, formation of skin lesions, oedema, and inflammation) differed significantly between experimental groups throughout all three experimental phases (Kruskal–Wallis test, *p* < 0.001).

In experimental groups treated exclusively with T-PC and not exposed to the carcinogen (Groups I–II), the formulation was well tolerated on intact skin, with no clinically relevant irritation observed even at the higher C-PC concentration.

During Phase I, the carcinogen control group (Group III) exhibited significantly higher skin irritation scores than both the T-PC-only groups (Groups I and II) and the T-PC-treated carcinogen groups (Groups V and VI) (adjusted *p* < 0.05). In Phase II, after modifying the TPA from an acetone vehicle to PEG/acetone, intergroup differences persisted but were less pronounced. The carcinogen control group (Group III) remained significantly different from the non-carcinogen groups, while no statistically significant differences were observed among the carcinogen-treated groups. In Phase III, the reintroduction of TPA in acetone led to a renewed increase in skin irritation severity, with the carcinogen control group (Group III) again exhibiting higher scores and significantly differing from the T-PC-treated carcinogen group (Group V).

**Figure 2:**
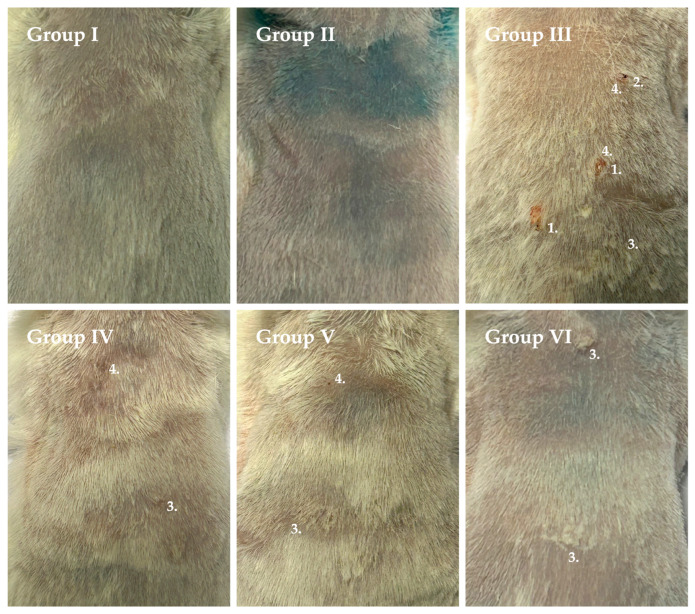
Representative macroscopic images of dorsal skin condition in mice from all experimental groups at the end of the study (week 16). (1) Papilloma-like lesions; (2) wound formation; (3) dryness and flaking; (4) signs of inflammation.

Representative macroscopic images of dorsal skin from all experimental groups at study termination (week 16) are presented in Figure 2.

At study termination, only a limited number of animals in the carcinogen control group (Group III) developed small superficial papilloma-like lesions, whereas the predominant macroscopic phenotype was inflammatory and irritative skin damage. Notably, carcinogen-treated groups receiving transfersome-based formulations exhibited visually improved skin condition compared to the carcinogen control group.

### 3.2. Histopathological Evaluation of Skin Tissue

#### 3.2.1. Epidermal Alterations

In this study, the primary focus was on evaluating epidermal alterations, including epidermal thickening, dysplasia, hyperplasia, oedema, and mitotic activity, as the development of fully established squamous cell carcinoma was not achieved in compliance with animal welfare regulations.

In Groups I and II, animals were not exposed to the carcinogen, and these groups were intended to demonstrate that T-PC, when applied topically to the skin, does not induce epidermal hyperplasia, dermal inflammation, or increased proliferative activity and, therefore, may be considered safe in the context of carcinogenesis modulation.

**Figure 3:**
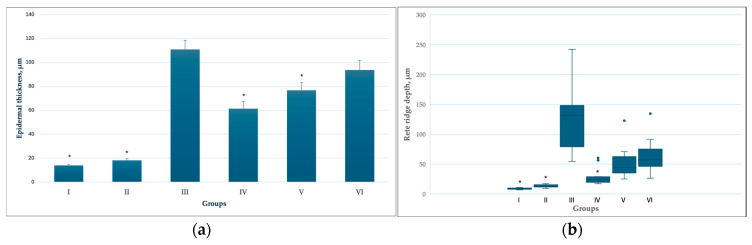
(**a**) Distribution of epidermal thickness across different experimental groups. Data are presented as mean and SD. * *p* < 0.05 compared with the carcinogen-only group (Group III), Games–Howell post hoc test following Welch’s ANOVA. (**b**) Distribution of rete ridge depth across different experimental groups. Data are shown as box-and-whisker plots displaying the median, interquartile range (IQR), and range; outliers are shown as individual points. Statistical analysis was performed using the Kruskal–Wallis test followed by Bonferroni-adjusted pairwise comparisons. * *p* < 0.05 versus the carcinogen-only group (Group III).

Epidermal thickness and rete ridge depth differed among the experimental groups (Figure 3a,b). The lowest values were observed in the control groups not exposed to the carcinogen (Groups I and II), in which epidermal architecture remained close to physiological conditions, with epidermal thickness ranging from 10 to 20 µm. In these groups, rete ridges were short and uniform, consistent with normal skin architecture (rete ridge depth 0–10 µm, predominantly flat rete ridges with a height of 3–4 nuclei).

In all carcinogen-exposed groups (Groups III, IV, V, and VI), a marked increase in epidermal thickness was observed, indicating proliferative and hyperplastic alterations of the epidermis. The greatest epidermal thickness was recorded in the carcinogen-alone group (Group III) and in the carcinogen combined with transfersomes containing a higher concentration of C-PC (Group VI). Although epidermal thickness in Groups IV and V was increased compared with the non-carcinogen control groups (Groups I and II), it was significantly lower than in the carcinogen-only group (Group III).

Analysis of rete ridge depth revealed a similar pattern. Carcinogen-exposed groups exhibited significant elongation of rete ridges, particularly in Group III, where deep epidermal invaginations into the dermis were observed. These changes indicate active remodelling of epidermal architecture characteristic of preneoplastic processes. Notably, rete ridge depth in Group IV was significantly lower than in the carcinogen-only group (Group III).

**Figure 4:**
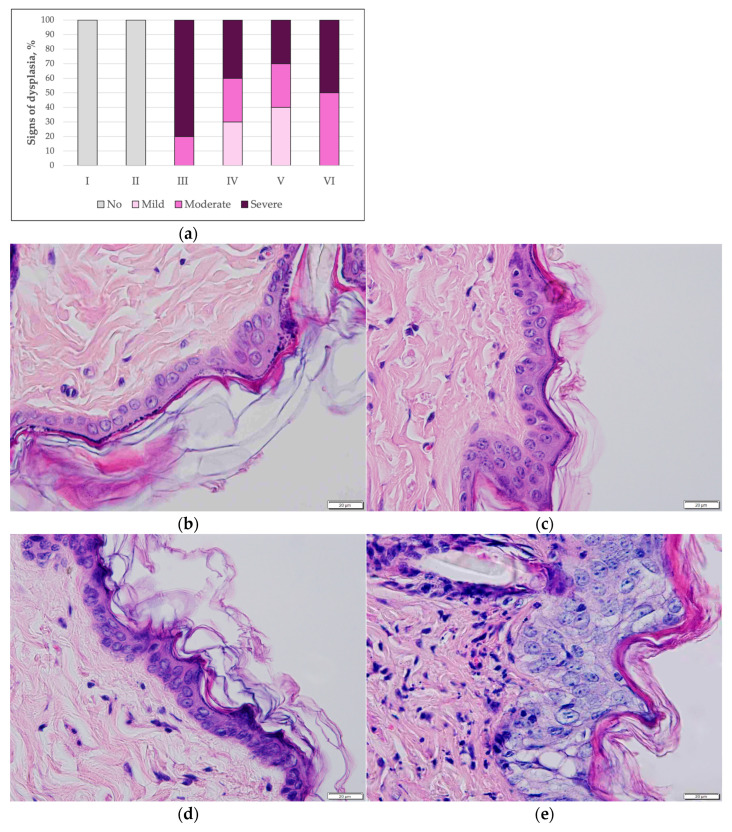

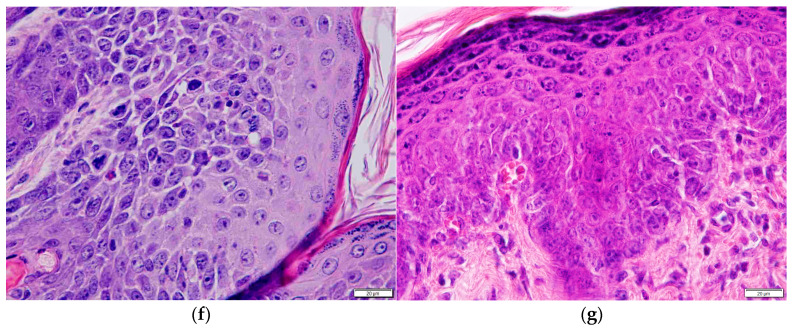
(**a**) Distribution of epidermal dysplasia grades across experimental groups. The percentage distribution of epidermal dysplasia grades is presented for each experimental group. (**b**) Histological appearance of the non-treated epidermis. H&E, ×200. (**c**) Histological appearance of the epidermis, T-PC 1 mg/mL (Group I). H&E, ×400. (**d**) Histological appearance of the epidermis, T-PC 10 mg/mL (Group II). H&E, ×400. (**e**) Histological appearance of the epidermis showing severe epidermal dysplasia (Group III): marked nuclear pleomorphism, hyperchromasia, and an increased nuclear-to-cytoplasmic (N/C) ratio are observed not only in the basal layer but also in the suprabasal layers; cellular differentiation is disrupted, cells are arranged irregularly, and the normal epidermal architecture is disturbed; no evidence of invasion through the basement membrane is observed. H&E, ×400. (**f**) Histological appearance of the epidermis showing moderate epidermal dysplasia (Group IV): pronounced nuclear pleomorphism is observed, characterized by variably sized, irregular, hyperchromatic nuclei and an increased nuclear-to-cytoplasmic ratio. Mitotic figures are present, some of which are observed not only in the basal layer. Epidermal layer differentiation is disturbed, with indistinct boundaries between the basal and parabasal layers and irregular arrangement of keratinocytes. However, cells with nuclear atypia are not yet present throughout all epidermal layers, and cells in the upper layers remain fully differentiated. H&E, ×400. (**g**) Histological appearance of the epidermis: mild epidermal dysplasia (Group V). Some keratinocyte nuclei are variably sized, irregularly shaped, and hyperchromatic. Mild disturbance of cellular polarity is observed in the lower epidermal layers, with an increased nuclear-to-cytoplasmic (N/C) ratio in some cells. Disruption of maturation is more pronounced in the lower third of the epidermis, while mature, well-differentiated cells are observed in the upper layers. H&E, ×400.

Epidermal dysplasia. The distribution of epidermal dysplasia grades differed among the experimental groups (Figure 4a). No epidermal dysplasia was detected in the groups not exposed to the carcinogen (Groups I and II), accounting for 0% of all cases (Figure 4c,d). In these groups, epidermal morphology was comparable to that of untreated mice maintained under identical conditions (Figure 4b). The epidermis remained close to physiological conditions, appearing very thin and composed of 2–3 layers of keratinocytes. Keratinocyte nuclei were regularly arranged, the stratum spinosum layer was minimal, and the stratum corneum was thin and compact, without evidence of hyperkeratosis or spongiosis. No epidermal oedema was observed.

In the group treated with the carcinogen in combination with empty transfersomes (Group IV), a mixed spectrum of dysplasia was observed, with mild dysplasia accounting for approximately 30%, moderate dysplasia for approximately 30%, and severe dysplasia for approximately 40%. In the group treated with the carcinogen together with T-PC at a concentration of 1 mg/mL (Group V), mild dysplasia accounted for approximately 40% of all cases, while the proportion of severe dysplasia decreased from approximately 80% to around 30% compared with the carcinogen-only group. In the group treated with the carcinogen combined with T-PC at a concentration of 10 mg/mL (Group VI), severe dysplasia accounted for approximately 50% of cases.

In contrast, in the group treated with the carcinogen alone (Group III), severe dysplasia predominated, accounting for approximately 80% of all cases, whereas moderate dysplasia was observed in only approximately 20% of animals. Representative histological examples of different grades of epidermal dysplasia are shown in Figure 4e–g.

**Figure 5:**
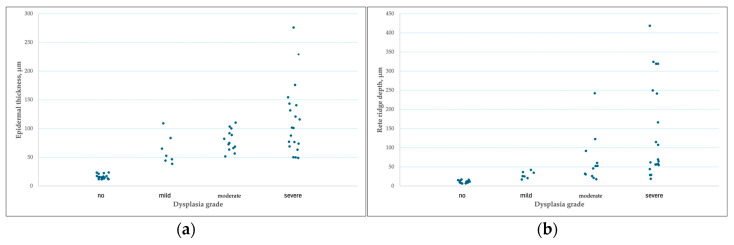
(**a**) Distribution of epidermal thickness across dysplasia grades. (**b**) Distribution of rete ridge depth across dysplasia grades. Dots represent individual measurements. A strong positive correlation with dysplasia grade was observed (Spearman’s ρ = 0.828 and 0.834, respectively; *p* < 0.001).

Both epidermal thickness and rete ridge depth showed strong positive correlations with dysplasia grade (Spearman’s ρ = 0.828 and 0.834, respectively, *p* < 0.001; Figure 5). A clear trend of increasing epidermal thickness with increasing dysplasia severity was observed, with lower dysplasia grades associated with a thinner epidermis, whereas higher-grade dysplasia was characterized by marked epidermal thickening. This relationship indicates that increased epidermal thickness represents a consistent feature of dysplastic progression and reflects structural remodelling of the epidermis accompanying preneoplastic processes.

Further analysis of rete ridge depth in relation to dysplasia grade demonstrated a similarly strong positive association, with increasing dysplasia severity corresponding to progressively elongated rete ridges. Higher-grade dysplasia was associated with deep, irregularly distributed rete ridges extending into the deeper dermal layers.

**Figure 6:**
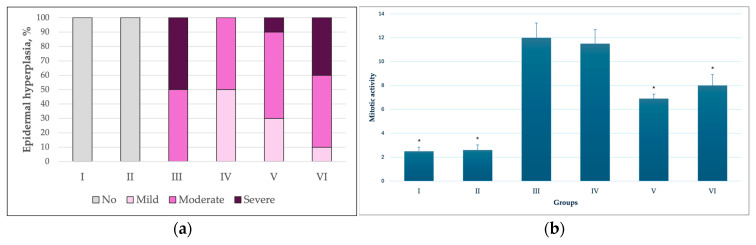
(**a**) Distribution of epidermal hyperplasia severity across experimental groups. The percentage distribution of epidermal hyperplasia grades is presented for each experimental group. (**b**) Distribution of epidermal mitotic activity (number of mitoses per 2.37 mm^2^) across experimental groups. Data are presented as mean and SD. * *p* < 0.05 compared with the carcinogen-only group (Group III), one-way ANOVA followed by Tukey’s post hoc test.

Hyperplasia. In the control groups (Groups I and II), no epidermal hyperplasia was observed (Figure 6a). In the group treated with the carcinogen alone (Group III), moderate-to-severe epidermal hyperplasia predominated. In animals treated with the carcinogen in combination with empty transfersomes (Group IV), epidermal hyperplasia was most frequently classified as mild or moderate.

When the carcinogen was applied together with T-PC at a concentration of 1 mg/mL (Group V), a reduced proportion of severe hyperplasia was observed, with mild and moderate hyperplasia predominating. In the group treated with the carcinogen combined with T-PC at 10 mg/mL (Group VI), moderate and severe epidermal hyperplasia still predominated; however, the overall severity was slightly lower than in the carcinogen-only group. The superior effect observed at the lower C-PC concentration may reflect formulation-dependent factors rather than purely pharmacological activity. Higher loading levels may reduce vesicle deformability or promote partial encapsulation saturation, potentially limiting dermal penetration efficiency. These findings indicate that treatment with transfersomes was associated with a reduction in the intensity of epidermal hyperplasia, although it did not eliminate hyperplastic changes.

Mitotic activity. Evaluation of mitotic activity, expressed as the number of mitoses per 2.37 mm^2^, revealed minimal proliferative activity in the control groups (Groups I and II), with an average of 2–3 mitoses per 2.37 mm^2^ (Figure 6b). In contrast, groups exposed to the carcinogen exhibited markedly increased mitotic activity, reaching up to approximately 12 mitoses per 2.37 mm^2^. Application of T-PC resulted in significantly lower mitotic activity compared with the carcinogen-only group and the group treated with the carcinogen combined with empty transfersomes.

**Figure 7:**
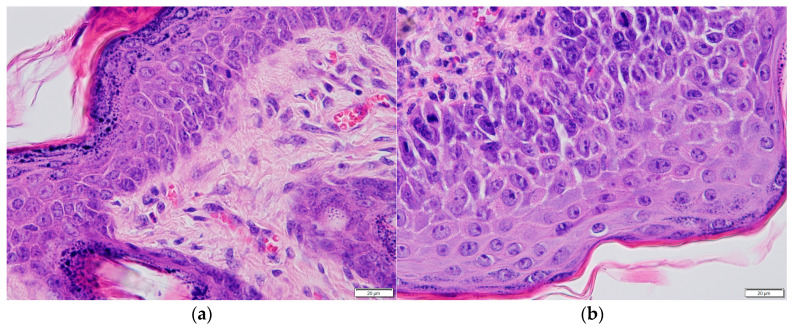
(**a**) Histological appearance of the epidermis showing mild oedema: slightly widened intercellular spaces between keratinocytes are visible, particularly within the stratum spinosum; keratinocytes remain normally interconnected (carcinogen + T-PC, Group V). H&E, ×400. (**b**) Histological appearance of the epidermis showing marked (moderate) oedema: clearly widened intercellular spaces between keratinocytes are observed, particularly within the stratum spinosum; however, keratinocytes remain interconnected (carcinogen group, Group III). H&E, ×400.

Epidermal oedema (spongiosis) was detected exclusively in the carcinogen-exposed groups and showed considerable variability both between and within the groups (Figure 7). In the group treated with the carcinogen alone (Group III), marked epidermal oedema predominated, characterized by widened intercellular spaces between keratinocytes and disruption of normal epidermal architecture.

In groups treated with T-PC and the carcinogen, epidermal oedema intensity ranged from mild to severe but did not show a consistent association with proliferative activity or dysplasia grade. No epidermal oedema was observed in the control groups.

The presence of both intercellular oedema (spongiosis) and intracellular oedema in histological sections suggests that the oedematous changes observed in this model most likely reflect acute carcinogen-induced tissue injury and an inflammatory response rather than a direct component of preneoplastic transformation.

#### 3.2.2. Dermal Alterations

**Figure 8:**
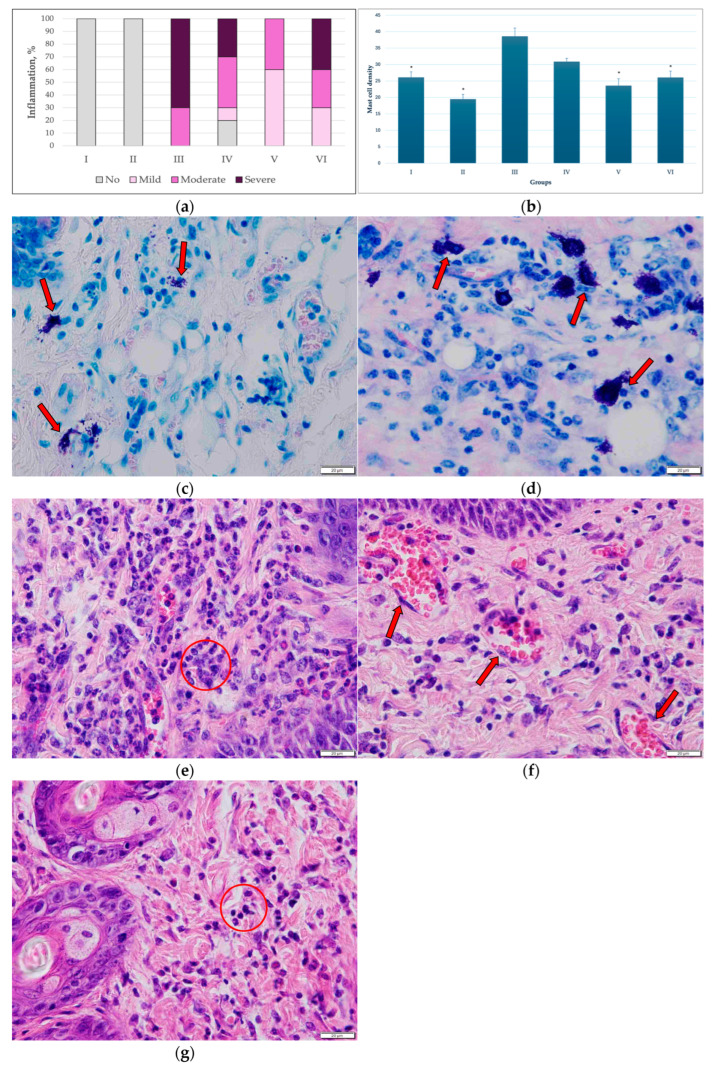
(**a**) Distribution of inflammation grades across different experimental groups, presented as percentage values. (**b**) Mast cell density (number of mast cells per 2.37 mm^2^) in the dermis of different experimental groups. Values are expressed as mean and SD. Statistical differences among groups were analysed using one-way ANOVA followed by Tukey’s multiple comparisons test (* *p* < 0.05 versus Group III). (**c**) Sparse mast cells (red arrows) in the dermis, Giemsa staining (Group V). (**d**) Numerous mast cells (red arrows) in the dermis, Giemsa staining (Group III). (**e**) Marked dermal inflammation characterized by abundant neutrophils (outlined in red), many exhibiting reactive (band-shaped) nuclei (Group III). (**f**) Pronounced dermal hyperemia associated with severe inflammation, showing dilated and blood-filled vessels (erythrocytes indicated by red arrows) (Group III). (**g**) Significantly reduced inflammatory response in the dermis, neutrophils outlined in red (Group V).

Inflammation. The distribution of dermal inflammation severity differed markedly among the experimental groups (Figure 8a).

No inflammatory changes were observed in the control groups (Groups I–II). In contrast, the group treated with the carcinogen alone (Group III) exhibited predominantly severe dermal inflammation (Figure 8e), accounting for the majority of cases (70%), with the remaining samples showing moderate inflammation. In the group treated with the carcinogen in combination with empty transfersomes (Group IV), the inflammatory response was more heterogeneous. While moderate and severe inflammation remained prevalent, some samples exhibited mild or no inflammation, suggesting partial attenuation of the inflammatory response compared with the carcinogen-only group. In the groups treated with the carcinogen together with T-PC (Groups V and VI), a clear shift toward lower inflammation severity was observed. In Group V (Figure 8g), mild inflammation predominated, with fewer cases of moderate and severe inflammation. Similarly, in Group VI, although moderate and severe inflammation remained present, their proportions were reduced compared with the carcinogen-only group, and mild inflammation accounted for a substantial fraction of cases.

Overall, these findings indicate that application of C-PC, particularly when delivered via transfersomes at a concentration of 1 mg/mL (Group V), was associated with a reduction in the intensity of dermal inflammation, shifting the inflammatory profile from predominantly severe toward milder forms, although inflammation was not completely abolished.

Hyperaemia was detected exclusively in the carcinogen-exposed groups (Figure 8f) and was closely associated with the intensity of the inflammatory infiltrate. No hyperaemia was observed in the control groups treated with T-PC only (Groups I and II). In the group treated with the carcinogen alone (Group III), moderate to severe hyperaemia predominated, whereas in the groups treated with the carcinogen and T-PC (Groups V and VI), mild to moderate hyperaemia was more frequently observed.

Oedema was evaluated as a reactive change and a component of inflammation, particularly acute inflammation. No oedema was observed in the control groups treated with T-PC only (Groups I and II). In the group exposed to the carcinogen alone (Group III), oedema was most often moderate to severe. In the groups in which the carcinogen was applied together with transfersomes, either empty or in combination with C-PC (Groups IV, V, and VI), the oedema intensity was lower, with mild to moderate oedema predominating and severe oedema observed less frequently. In all carcinogen-exposed groups (Groups III, IV, V, and VI), oedema correlated with the intensity of inflammation and was interpreted as a secondary tissue response rather than an independent pathological process.

Mast cell density was lower in the groups not exposed to the carcinogen and differed significantly among the carcinogen-exposed groups (Figure 8b–d). The highest mast cell density was observed in the carcinogen-only group (Group III), indicating a pronounced activation of the dermal inflammatory microenvironment. In the groups treated with the carcinogen in combination with empty transfersomes (Group IV), mast cell density remained elevated but was lower than in the carcinogen-only group, suggesting partial attenuation of the inflammatory response. In the groups treated with the carcinogen together with T-PC (Groups V and VI), mast cell density was significantly reduced compared with the carcinogen-only group. Among these, Group V exhibited the lowest mast cell density, while Group VI showed slightly higher values, although still significantly lower than those observed in Group III.

Overall, these findings indicate that T-PC application was associated with reduced mast cell accumulation in the dermis, suggesting a potential modulatory effect of C-PC on the inflammatory microenvironment induced by the carcinogen.

A significant positive correlation was found between dermal inflammation severity and mast cell density (Spearman’s ρ = 0.438, *p* < 0.001). Samples exhibiting more pronounced, diffuse inflammatory infiltrates showed higher mast cell counts, whereas in the control groups, only occasional lymphocytes were observed.

### 3.3. Immunohistochemical Assessment of Epidermal Proliferation and Its Association with Histopathological Parameters

**Figure 9:**
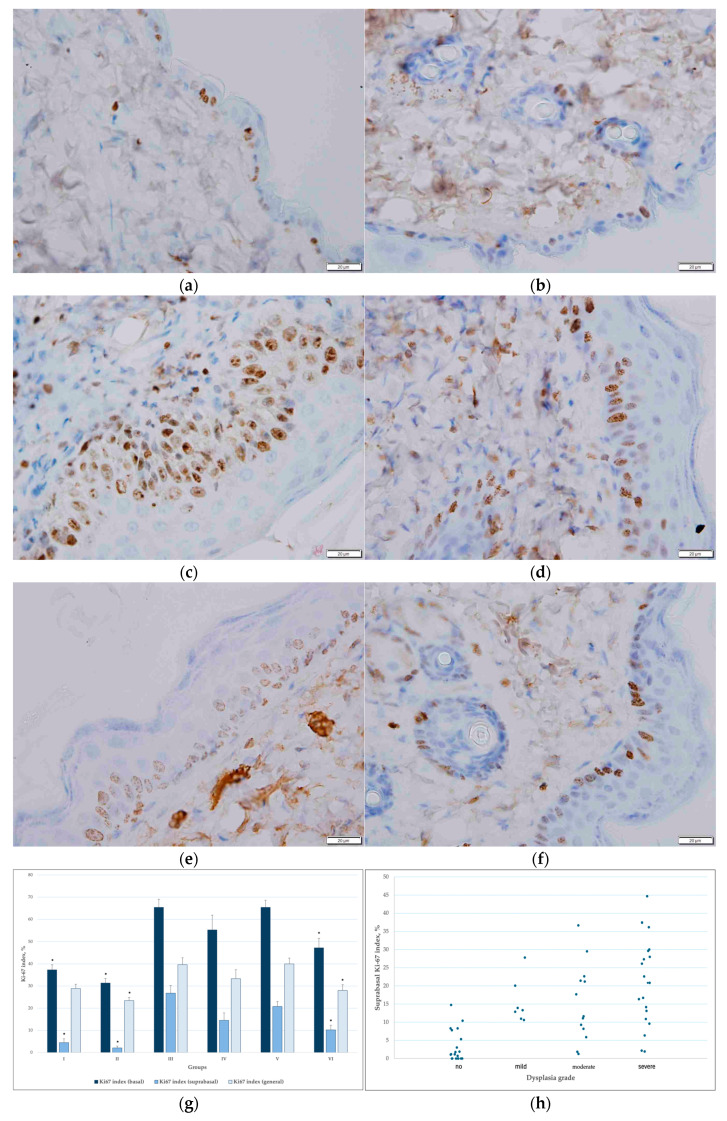

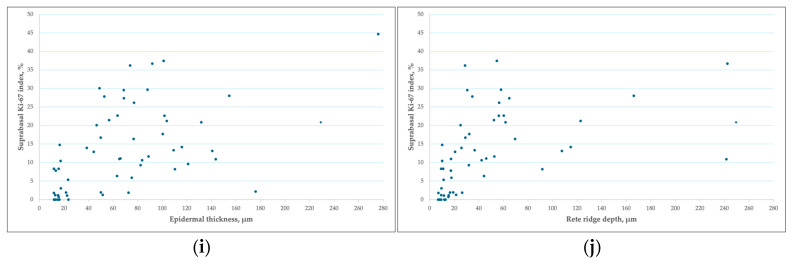
(**a**–**f**) Representative immunohistochemical staining of Ki-67 expression in the epidermis of experimental groups: (**a**) Group I, (**b**) Group II, (**c**) Group III, (**d**) Group IV, (**e**) Group V, (**f**) Group VI. (**g**) Ki-67 proliferation index in the epidermis across experimental groups. Data are presented as mean and SD for basal, suprabasal, and total epidermal Ki-67 indices. Statistical analysis was performed using one-way ANOVA with Tukey’s post hoc test for variables with homogeneous variances, and Welch’s ANOVA followed by Games–Howell post hoc test for variables with unequal variances. * *p* < 0.05 compared with the carcinogen-only group (Group III). (**h**) Relationship between epidermal dysplasia grade and epidermal proliferative activity. The scatter plot shows the epidermal Ki-67 proliferation index (%) for individual animals by dysplasia grade (no dysplasia, mild, moderate, severe). A positive correlation was observed (Spearman’s ρ = 0.66, *p* < 0.05). (**i**) Correlation between epidermal thickness and suprabasal Ki-67 index. The scatter plot illustrates the relationship between epidermal thickness and the suprabasal Ki-67 proliferation index (Spearman’s ρ = 0.64, *p* < 0.05). (**j**) Correlation between rete ridge depth and suprabasal Ki-67 index. The scatter plot illustrates the relationship between rete ridge depth and the suprabasal Ki-67 proliferation index (Spearman’s ρ = 0.71, *p* < 0.05).

Quantitative analysis of epidermal proliferation revealed significant differences in Ki-67 expression among the experimental groups (Figure 9a–g). In the basal epidermal layer, the Ki-67 index was significantly higher in the carcinogen-treated group (Group III) compared with the control groups (Groups I and II) (*p* < 0.05). Treatment with T-PC resulted in a partial reduction in basal proliferative activity, with significant differences observed between Group III and Group VI (*p* < 0.05), whereas differences between Group III, Group IV, and Group V were less consistent.

Suprabasal Ki-67 index was markedly increased in the carcinogen-only group (Group III) compared with both control groups (Groups I and II) (*p* < 0.05). Application of empty transfersomes or T-PC reduced suprabasal proliferative activity, especially in Group VI relative to Group III (*p* < 0.05), although values remained elevated compared with the controls. These findings indicate that suprabasal Ki-67 expression is a particularly sensitive marker of pathological epidermal proliferation.

Analysis of the total epidermal Ki-67 index also revealed a statistically significant reduction in carcinogen and T-PC-treated group (Group VI) compared with the carcinogen-only group (Group III, (*p* < 0.05)), while the other carcinogen-and transfersomes-treated groups did not show statistical significance. Overall, the total Ki-67 index was less sensitive to intergroup differences than separate analyses of the basal and suprabasal epidermal layers.

Although occasional Ki-67-positive cells were observed in the suprabasal layer of the control groups treated with T-PC-only, this finding likely reflects physiological epidermal regeneration and mild mechanical or chemical irritation associated with topical application, animal scratching behaviour, or minor microtrauma due to the skin shaving process, rather than pathological proliferative activity.

As the suprabasal Ki-67 index was identified as a more sensitive marker of pathological proliferation, correlation analysis was performed between suprabasal Ki-67 expression and selected histopathological parameters, including dysplasia grade, epidermal thickness, and rete ridge depth.

The strongest statistically significant positive association was observed between epidermal proliferative activity and rete ridge depth (Spearman’s ρ = 0.71, *p* < 0.05, Figure 9j). A positive correlation between the Ki-67 indexes and epidermal thickness was also found (Spearman’s ρ = 0.64, *p* < 0.05, Figure 9i). Epidermal thickness and rete ridge depth reflect structural alterations in epidermal architecture that develop during carcinogenesis, not only because of increased basal-layer proliferation, but also because proliferative activity expands into the suprabasal epidermal layers. A positive correlation between epidermal dysplasia grade and epidermal proliferative activity (Spearman’s ρ = 0.66, *p* < 0.05, Figure 9h) indicated that higher dysplasia severity in this experimental model was associated with increased epidermal proliferative activity.

Immunohistochemical analysis of proliferation marker (Ki-67) showed extensive nuclear positivity in basal and suprabasal epidermal layers in the carcinogen group (Group III). In contrast, C-PC treatment significantly reduced the number of Ki-67–positive cells in a dose-dependent manner, indicating suppressed epidermal cell proliferation.

### 3.4. Histopathological Assessment of Internal Organs

Histological evaluation of internal organs, including liver, spleen, lungs, heart, kidneys, brain, testes, and mesenteric lymph nodes, revealed no treatment-related pathological alterations in any experimental group.

All examined organs maintained normal histoarchitecture, with no evidence of degenerative, necrotic, inflammatory, or neoplastic changes, confirming the systemic safety of topically applied C-PC-loaded transfersomes.

## 4. Discussion

The present study was designed as a proof-of-concept investigation to evaluate the preventive potential of C-PC-loaded transfersomes in the early stages of skin carcinogenesis using the widely established DMBA/TPA mouse model proposed by Li et al. [17]. A single application of DMBA induces oncogenic mutations—most commonly in *Hras*—whereas repeated TPA treatment promotes inflammation-driven clonal expansion and progression toward squamous cell carcinoma. Importantly, this mouse model exhibits genomic similarities to several subtypes of human SCC, supporting its translational relevance [17,20].

In the present study, carcinogenesis was initiated by a single topical application of DMBA, followed by twice-weekly application of TPA to promote tumour development. TPA is considered one of the most powerful skin tumour promoters and exerts its effects by profoundly reshaping molecular pathways involved in tumour promotion. By stimulating clonal expansion of initiated cells, TPA induces a strong inflammatory response while suppressing programmed cell death, thereby creating a microenvironment favourable for tumour progression. At the molecular level, TPA-induced upregulation of ornithine decarboxylase (ODC), cyclooxygenase-2 (COX-2), and interleukin-6 (IL-6), together with activation of the IL-6/STAT3 signalling axis and suppression of transglutaminase-2 (TG2), plays a central role in epidermal hyperproliferation, inflammation, and tumour promotion [21].

Although the DMBA/TPA model is widely used to induce cSCC, progression to fully developed invasive cSCC was not achieved under the present experimental conditions in accordance with animal welfare requirements. Therefore, this study focused primarily on early tumour-promoting and preneoplastic epidermal alterations rather than on overt tumour burden or malignant progression. Importantly, these early inflammation-driven changes represent a biologically relevant stage of carcinogenesis, during which modulation of the epidermal microenvironment may critically influence subsequent malignant transformation.

Consistent with this concept, pronounced precancerous histopathological alterations were observed in the carcinogen control group (Group III). The observed changes were characteristic of inflammation-driven tumour promotion in the DMBA/TPA model, including pathways associated with COX-2–IL-6–STAT3 signalling. Group III exhibited predominantly severe dermal inflammation (70% of cases), accompanied by hyperaemia, oedema, and increased mast cell density, indicating marked activation of the dermal inflammatory microenvironment. In parallel, substantial epidermal remodelling was evident, including pronounced epidermal thickening, increased rete ridge depth, and a predominance of moderate-to-severe hyperplasia. Severe epidermal dysplasia was detected in 80% of samples, with the remaining cases showing moderate dysplasia. These structural alterations were further supported by significantly elevated proliferative activity, as demonstrated by increased mitotic counts (up to ~12 mitoses per 2.37 mm^2^) and a markedly elevated Ki-67 index.

Cutaneous squamous cell carcinoma develops through a well-recognized multistep process characterized by progressive architectural and molecular alterations of the epidermis. Disease progression typically evolves from actinic keratosis (AK) and intraepidermal dysplasia toward invasive carcinoma, accompanied by increasing keratinocyte proliferation, impaired differentiation, and chronic inflammation. Approximately 70% of cSCC cases are estimated to arise from pre-existing AK lesions, highlighting AK as a clinically relevant precursor stage [1,2]. Histologically, AK represents a continuous dysplastic spectrum ranging from basal-layer atypia to a full-thickness intraepidermal disorder consistent with SCC in situ, frequently associated with hyperkeratosis and dyskeratosis [1]. The epidermal disorganization, keratinocyte atypia, and hyperproliferative features observed in the present study are consistent with these early carcinogenic stages rather than fully developed invasive carcinoma. Given that cSCC arises through progressive precancerous stages, early intervention aimed at attenuating inflammation-and proliferation-driven events represents a rational preventive strategy, particularly in high-risk populations. Algae-derived bioactive compounds have attracted increasing attention in this context. Notably, extracts of *Limnospira platensis* (formerly *Spirulina platensis*), rich in C-PC, have demonstrated inhibitory effects in experimental models of chemically induced carcinogenesis [20]. Therefore, this study evaluated whether C-PC-loaded transfersomes could interfere with early tumour-promoting alterations in the skin.

Based on our previous ex vivo skin penetration studies, the free C-PC solution showed undetectable fluorescence within the skin layers, indicating limited skin penetration due to its high molecular weight and hydrophilic nature. In contrast, C-PC-loaded transfersomes significantly enhanced epidermal accumulation of C-PC [14]. Therefore, the present in vivo study was designed to evaluate the biological effects of the optimized transfersomal formulation, while groups treated with empty transfersomes were included to distinguish the effects of the carrier system from those of the encapsulated active compound. Evaluation of the study results demonstrated that transfersomes, when used with the carcinogen, markedly improved histopathological conditions in mouse skin by attenuating the development of hyperplasia and dysplasia, reducing epidermal thickening, and suppressing inflammatory and proliferative processes. Notably, even empty transfersomes (without C-CP) exerted a protective effect: severe hyperplasia was completely prevented, severe dysplasia was reduced by up to 40% compared with the carcinogen-only group, and the proportion of severe inflammation decreased from 70% to 30%. This suggests that transfersomes may modulate cutaneous responses not only through active compound delivery but also through carrier-dependent mechanisms, such as improved stratum corneum hydration, modulation of the lipid microenvironment, and barrier stabilization. Recent studies demonstrated that improved barrier integrity, together with modulation of inflammatory pathways, contributes to overall treatment outcomes [22]. In this context, the carrier-dependent effects observed in the present study may similarly support the therapeutic response.

Glycerol, a component of the transfersomal formulation, may contribute to the observed biological effects not only through its well-established role as a humectant but also via anti-inflammatory mechanisms. In an imiquimod-induced murine model of psoriasiform dermatitis, topical glycerol significantly reduced erythema, inflammation, and epidermal thickness [23]. Moreover, glycerol may indirectly support skin homeostasis by modulating the cutaneous microbiome, as commensal bacteria can ferment glycerol into bioactive metabolites, such as lactic acid, that inhibit pathogenic species, including *Staphylococcus aureus*, and upregulate genes associated with barrier function [24]. Glycerol plays a central role in maintaining epidermal hydration and barrier integrity, with high concentrations present in the stratum corneum. Glycerol generated through lipolysis and transported via AQP3 in the basal and spinous layers is essential for maintaining these elevated levels, whereas altered AQP3 expression has been linked to psoriasis, non-melanoma skin cancer, and atopic eczema [25]. Thus, exogenous glycerol delivered via transfersomes may support barrier stabilization and, by indirect mechanisms, attenuate inflammation-driven epidermal hyperproliferation observed in the DMBA/TPA model.

When discussing the effects of C-PC, it is essential to consider that in Groups I and II, where T-PC was applied to intact skin without carcinogen exposure, the formulation was well tolerated and did not induce clinically relevant irritation, even at the higher C-PC concentration. Histopathological analysis revealed preserved epidermal architecture with physiological thickness, short, uniform rete ridges, and normally arranged keratinocytes. No epidermal hyperplasia, dysplasia, spongiosis, oedema, hyperaemia, or inflammatory infiltrates were observed. These findings indicate that topical T-PC does not induce structural or proliferative alterations in healthy skin and may be considered safe under non-carcinogenic conditions.

Previous studies demonstrate that C-PC suppresses TPA-induced skin carcinogenesis pathways in a dose-dependent manner by reducing ODC, COX-2, and IL-6 expression and inhibiting STAT3 phosphorylation, while simultaneously inducing TG-2 expression, which is associated with differentiation and apoptosis [21]. In vivo studies further showed that C-PC significantly inhibits DMBA-induced skin tumour development by decreasing tumour incidence and size, delaying tumour onset, and modulating key regulators of the cell cycle and apoptosis, including mutant p53, Cdc25A, Bcl-2, and p27 [13]. Beyond its anti-proliferative effects, C-PC exhibits pronounced antioxidant and anti-inflammatory properties, including inhibition of NF-κB activation, suppression of NADPH oxidase, and activation of the Nrf2/HO-1 pathway [26,27]. While systemic anti-inflammatory effects are largely attributed to hepatic metabolites such as phycocyanorubin, topical delivery models indicate that C-PC also exerts local antioxidant and anti-inflammatory actions. Indeed, recent evidence demonstrates that topically administered C-PC directly suppresses NF-κB signalling, reduces pro-inflammatory cytokine expression, attenuates oxidative stress, and promotes M2 macrophage polarization within the skin microenvironment [9,27]. Modulation of the tumour microenvironment represents a critical component of cutaneous squamous cell carcinoma (CSCC) progression [28]. Chronic inflammation, COX-2 overexpression, stromal activation, and immune cell infiltration contribute to tumour promotion and angiogenesis [29].

In the present study, C-PC-loaded transfersomes—particularly at 1 mg/mL—markedly reduced skin inflammation and structural epidermal alterations, including epidermal thickness and rete ridge depth. The more pronounced effect observed at the lower concentration may indicate that optimal biological performance depends on preserving vesicle elasticity rather than maximal drug loading. Higher C-PC content may alter membrane packing and reduce transfersome flexibility, thereby limiting penetration efficiency. Both C-PC concentrations significantly decreased mast cell density. Previous studies have shown that mast cells are key regulators of the tumour microenvironment. Upon activation, they release a wide range of pro-inflammatory and pro-angiogenic mediators, including vascular endothelial growth factor (VEGF), fibroblast growth factor-2 (FGF-2), tumour necrosis factor α (TNF-α), transforming growth factor-β, interleukins (IL1, IL3, IL4, IL5, IL6 IL8, IL10), and matrix metalloproteinases, which contribute to angiogenesis, extracellular matrix remodelling, and tumour progression [30,31,32]. Moreover, mast cell density has been shown to increase during disease progression from dysplasia to SCC [32]. These findings are in line with previous reports from DMBA/croton oil-induced mouse models of skin tumorigenesis, where mast cell density was significantly increased in carcinogen-treated animals, whereas treatment with C-type natriuretic peptide, alone or in combination with cisplatin, reduced mast cell infiltration [33]. Therefore, the observed reduction in mast cell density in C-PC-loaded transfersome-treated groups may contribute to the suppression of inflammation-driven epidermal remodelling. However, this interpretation remains indirect, as angiogenic markers and specific inflammatory mediators were not directly assessed in the present study. Furthermore, both C-PC concentrations significantly reduced mitotic activity, while the higher concentration (10 mg/mL) additionally decreased basal and suprabasal Ki-67 proliferation indexes. Taken together, the observed effects likely result from the combined effects of enhanced dermal delivery and carrier-mediated modulation of the skin microenvironment. Collectively, these findings support the role of C-PC-loaded transfersomes in attenuating inflammation-driven epidermal remodelling and suppressing early tumour-promoting events.

Despite the promising findings, several limitations of the present study should be acknowledged. First, only male animals were included in the experimental design. Since sex-related differences in skin physiology, hormonal regulation, and inflammatory responses may influence carcinogenesis and treatment outcomes, future studies including both sexes would further strengthen the generalizability of these findings. Furthermore, the present study focused exclusively on the protective effects of C-PC-loaded transfersomes during early stage of cSCC development. In later stages of tumour progression, the tumour microenvironment becomes increasingly complex, with enhanced angiogenesis, immune evasion, and invasive behaviour [2]. Given that C-PC has been reported to promote pro-apoptotic signalling while suppressing anti-apoptotic pathways, it may also possess therapeutic potential in more advanced stages of cSCC development [34]. Therefore, additional investigations evaluating the effects of C-PC-loaded transfersomes during later stages of carcinogenesis, including advanced dysplastic lesions and invasive squamous cell carcinoma, are warranted. In addition, the formulation was not directly compared with an established standard topical therapeutic agent, which should be addressed in future studies to further evaluate its relative efficacy and clinical potential.

## 5. Conclusions

The present study demonstrates that topical C-PC-loaded transfersomes attenuate early carcinogenesis-associated epidermal and dermal alterations in the DMBA/TPA mouse model. C-PC delivery significantly reduced inflammation severity, mast cell accumulation, mitotic activity, and basal and suprabasal Ki-67 expression, indicating suppression of pathological proliferation and tumour-promoting epidermal remodelling. These findings further suggest that transfersomal systems may modulate early tumour-promoting events not only by enhancing the delivery of C-PC but also, potentially, through carrier-related physicochemical interactions with the cutaneous barrier.

Further studies investigating molecular signalling pathways and long-term tumour incidence are warranted to validate the potential clinical applicability of C-PC-loaded transfersomes.

## Figures and Tables

**Table 1:** Experimental groups and treatment schedule.

Treatment	Group I	Group II	Group III	Group IV	Group V	Group VI
Group description	Non-carcinogen+ T-PC 1 mg/mL	Non-carcinogen+ T-PC 10 mg/mL	Carcinogen control	Carcinogen+ empty transfersomes	Carcinogen+ T-PC 1 mg/mL	Carcinogen+ T-PC 10 mg/mL
T-PC 1 mg/mL, (200 µL)	Twice/week	_	_	_	Twice/week	_
T-PC 10 mg/mL, (200 µL)	_	Twice/week	_	_	_	Twice/week
Empty transfersomes (200 µL)	_	_	_	Twice/week	_	_
DMBA (50 µg in acetone, 200 µL)	_	_	Single dose	Single dose	Single dose	Single dose
TPA (5 µg in acetone or PEG/acetone, 200 µL)	_	_	Twice/week	Twice/week	Twice/week	Twice/week

## Data Availability

The data supporting the findings of this study are available from the corresponding author (D.G.) upon reasonable request.
